# PERT: A Method for Expression Deconvolution of Human Blood Samples from Varied Microenvironmental and Developmental Conditions

**DOI:** 10.1371/journal.pcbi.1002838

**Published:** 2012-12-20

**Authors:** Wenlian Qiao, Gerald Quon, Elizabeth Csaszar, Mei Yu, Quaid Morris, Peter W. Zandstra

**Affiliations:** 1 Institute of Biomaterials and Biomedical Engineering, University of Toronto, Toronto, Ontario, Canada; 2 Department of Computer Science, University of Toronto, Toronto, Ontario, Canada; 3 Department of Chemical Engineering and Applied Chemistry, University of Toronto, Toronto, Ontario, Canada; 4 Department of Molecular Genetics, University of Toronto, Toronto, Ontario, Canada; 5 Banting and Best Department of Medical Research, University of Toronto, Toronto, Ontario, Canada; 6 Donnelly Centre for Cellular and Biomolecular Research, University of Toronto, Toronto, Ontario, Canada; 7 McEwen Centre for Regenerative Medicine, University of Health Network, Toronto, Ontario, Canada; 8 Heart & Stroke/Richard Lewar Centre of Excellence, Toronto, Ontario, Canada; New York University, United States of America

## Abstract

The cellular composition of heterogeneous samples can be predicted using an expression deconvolution algorithm to decompose their gene expression profiles based on pre-defined, reference gene expression profiles of the constituent populations in these samples. However, the expression profiles of the actual constituent populations are often perturbed from those of the reference profiles due to gene expression changes in cells associated with microenvironmental or developmental effects. Existing deconvolution algorithms do not account for these changes and give incorrect results when benchmarked against those measured by well-established flow cytometry, even after batch correction was applied. We introduce PERT, a new probabilistic expression deconvolution method that detects and accounts for a shared, multiplicative perturbation in the reference profiles when performing expression deconvolution. We applied PERT and three other state-of-the-art expression deconvolution methods to predict cell frequencies within heterogeneous human blood samples that were collected under several conditions (uncultured mono-nucleated and lineage-depleted cells, and culture-derived lineage-depleted cells). Only PERT's predicted proportions of the constituent populations matched those assigned by flow cytometry. Genes associated with cell cycle processes were highly enriched among those with the largest predicted expression changes between the cultured and uncultured conditions. We anticipate that PERT will be widely applicable to expression deconvolution strategies that use profiles from reference populations that vary from the corresponding constituent populations in cellular state but not cellular phenotypic identity.

## Author Summary

The cellular composition of heterogeneous samples can be predicted from reference gene expression profiles that represent the homogeneous, constituent populations of the heterogeneous samples. However, existing methods fail when the reference profiles are not representative of the constituent populations. We developed PERT, a new probabilistic expression deconvolution method, to address this limitation. PERT was used to deconvolve the cellular composition of variably sourced and treated heterogeneous human blood samples. Our results indicate that even after batch correction is applied, cells presenting the same cell surface antigens display different transcriptional programs when they are uncultured versus culture-derived. Given gene expression profiles of culture-derived heterogeneous samples and profiles of uncultured reference populations, PERT was able to accurately recover proportions of the constituent populations composing the heterogeneous samples. We anticipate that PERT will be widely applicable to expression deconvolution strategies that use profiles from reference populations that vary from the corresponding constituent populations in cellular state but not cellular phenotypic identity.

## Introduction

Heterogeneity as a description of a biological sample typically refers to the co-existence of phenotypically and functionally distinct cell populations therein. In a dynamic system such as *in vitro* stem cell growth and differentiation, cells continuously self-renew, differentiate and die in response to a changing microenvironment. The ability to elucidate compositions of heterogeneous samples with respect to their constituent (homogeneous) populations is a pre-requisite for identifying the parameters governing these dynamic systems. Although cellular compositions can be deconvolved using flow cytometry gated on constituent population-associated surface antigens or fluorescent intracellular proteins, these approaches are constrained by their requirements for sample formats – only cells in suspension media can be analysed – and have limited power to discover novel populations emerging from heterogeneous samples. A more efficient, unbiased cellular decomposition technique that recapitulates flow cytometry-based deconvolution of heterogeneous samples using less material is desirable.

For elucidating compositions of highly heterogeneous samples, gene expression-based cellular deconvolution is more efficient, unbiased and economical. The technique has been used to decompose samples from yeast cell culture [1], tumor tissues [2], and peripheral blood of systemic lupus erythematosus [3] and multiple sclerosis patients [4]. Existing studies model gene expression profiles of heterogeneous samples (termed mixed profiles) as positively weighted sums of the gene expression profiles of pre-specified reference populations, where these reference profiles are chosen to represent constituent populations within the heterogeneous samples. The task is to estimate the proportion of each reference population within the heterogeneous samples. These models have two major limitations. First, reference profiles for all constituent populations of the heterogeneous samples of interest have to be available; however, new cell types or populations may have emerged from cell differentiation in dynamic circumstances, and cannot be accounted for by existing methods. Second, reference profiles must accurately represent the gene expression profiles of the actual constituent populations (termed the constituent profiles) of the heterogeneous samples of interest. However, because reference population samples and heterogeneous samples of interest are likely collected separately and therefore may exhibit transcriptional variations due to microenvironmental (e.g., inter-cellular communication) and developmental (e.g., culture conditions) changes, reproduction of flow cytometry analysis under such transcriptional variations cannot be achieved by existing methods. Thus, we aimed to develop flexible deconvolution models that consider the presence of new cell types or populations in heterogeneous samples, and also consider systematic fluctuations in gene expression between reference profiles and constituent profiles.

Recently, Quon and Morris developed ISOLATE [5] based on the Latent Dirichlet Allocation (LDA) model [6] for estimating proportions of cancer cells in tumor samples using quantitative gene expression data. In contrast to the linear regression models, these models use a multinomial noise model [7] that is a better fit to measurement noise in gene expression data [8]. We hypothesized that these models could be extended to allow transcriptional variations between reference and constituent populations.

Here we compare four models: a linear regression model called the non-negative least squares model (NNLS) [9], the non-negative maximum likelihood model (NNML), the non-negative maximum likelihood new population model (NNML_np_), and the perturbation model (PERT). NNLS assumes all constituent populations are represented in the reference profiles, and uses a linear regression framework to estimate the proportion of each heterogeneous sample attributable to each of the reference populations. NNML makes the same assumptions and solves the same problem as NNLS, but uses the LDA [6] framework for posing and solving the problem. NNML_np_ is a version of ISOLATE [5] that assumes there is an additional constituent population in the heterogeneous samples that is not represented by the available reference profiles, and is therefore estimated. PERT is our new model that is based on the NNML framework but accounts for transcriptional variations between reference and constituent profiles. The models were applied to uncultured mono-nucleated and lineage-depleted (Lin-, where cells expressing blood cell lineage-associated cell surface antigens are removed) cells enriched from fresh human umbilical cord blood, and cultured-derived Lin- cells. Model predictions were validated using an established flow cytometry assay. Overall, our analysis demonstrated that averaged absolute differences between PERT's predictions and flow cytometry measurements were significantly lower than the other models for uncultured mono-nucleated cells, uncultured Lin- cells, and culture-derived Lin- cells. Gene Ontology enrichment analysis of the genes that underwent 2-fold perturbation when comparing uncultured with culture-derived cells suggested that the transcriptional variations between these two cell populations were the result of up-regulation of cell cycle related genes in culture-derived cells.

We show that (i) cells presenting the same cell surface antigens can exhibit differences in transcriptional programs when they are subjected to different microenvironmental and developmental conditions; (ii) these variations cannot be corrected using current batch effect models, highlighting the need for care when comparing primary cells subjected to different exogenous perturbations; and (iii) these variations can be captured by modeling a shared gene-specific rescaling (in other words, a multiplicative perturbation) as part of the expression deconvolution. Our new model, PERT, is a deconvolution model that addresses transcriptional variations between reference and constituent profiles. The model is readily applicable to circumstances where available reference profiles are collected under different microenvironmental or developmental conditions from the heterogeneous samples.

## Results

### Deconvolution model formulation

In this study, four models, NNLS, NNML, NNML_np_ and PERT, were compared for their ability to deconvolve uncultured and culture-derived heterogeneous human blood samples. We used two measures of success: deconvolution accuracy defined as the proportion of variance (R^2^) in the measured proportions of constituent populations explained by the model's predictions, and averaged absolute difference between model predictions and experimental measurements.

**Figure 1:**
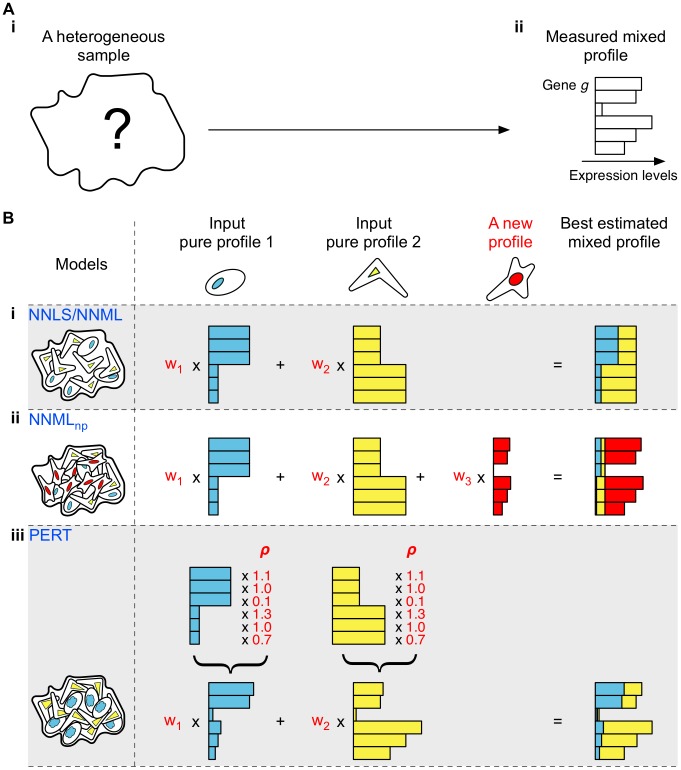
Schematic of deconvolution models. (A) Generation of mixed profiles from heterogeneous samples. (A-i) represents a heterogeneous sample whose composition is unknown. Each bar in (A-ii) represents individual gene expression levels of the heterogeneous sample. (B) Schematic of four deconvolution models. (B-i) The non-negative least squares model (NNLS) (Lawson and Hanson (1995)) and the non-negative maximum likelihood model (NNML) predict proportions of pre-specified reference populations in a heterogeneous sample using mixed and reference profiles. (B-ii) The non-negative maximum likelihood new population model (NNML_np_) estimates the gene expression profile of a new reference population that may exist in a heterogeneous sample; simultaneously, the model predicts proportions of both input reference populations and the new reference population. (B-iii) The perturbation model (PERT) perturbs the input reference profiles using a genome-wide perturbation vector ***ρ***; simultaneously, the model predicts proportions of the reference populations in a heterogeneous sample. Parameters shown in red are model predicted.

Given the gene expression profile of a heterogeneous sample that is a physical mixture of its constituent populations (Figure 1A), NNLS (Figure 1B-i) assumes that both the reference populations (whose gene expression profiles were provided for deconvolution) and the constituent populations were subjected to the same microenvironmental and developmental conditions and thus were equivalent. Therefore, a mixed profile is modeled as a positively weighted sum of reference profiles. Weight w_i_ indicates the proportion of reference population *i* within the heterogeneous sample, and is fit by minimizing the least squares error between the estimated and observed mixed profiles under an additive Gaussian measurement noise model [1], [3], [4], [10] while constraining the weights to be non-negative [9]. However, several studies have shown that the variance in gene expression measurement noise scales with the mean [8], [11], [12], contrary to the assumption of the additive Gaussian noise model. NNML [6] (Figure 1B-i) is similar to NNLS, but replaces the additive Gaussian measurement noise model with a multinomial noise model which has the desired scaling. However, neither NNLS nor NNML is designed to address two key challenges: first, the presence of additional constituent populations in the heterogeneous sample whose corresponding reference profiles are not available; second, transcriptional variations between constituents and corresponding reference populations that arise due to microenvironmental or developmental factors.

We addressed the first challenge using NNML_np_ (Figure 1B-ii). The model estimates the gene expression profile ***γ*** of a new, latent reference population to capture expression patterns in the heterogeneous samples that are not explained by the provided reference profiles. Simultaneously, the model estimates the proportions of individual reference populations in the heterogeneous samples.

We developed PERT (Figure 1B-iii) to address the second challenge. The model estimates a genome-wide perturbation vector ***ρ*** where each element of ***ρ***, *ρ_g_*, reflects the fold difference in expression of gene *g* in the constituent profiles versus the reference profiles: *ρ_g_*>1 indicates increased expression of gene *g* in constituent profiles compared to the reference profiles; *ρ_g_* = 1 indicates no change; and *ρ_g_*<1 indicates decreased expression. Simultaneously, the model estimates the proportions of individual reference populations in the heterogeneous samples (Materials and Methods).

### NNML does not require cell line signature genes

**Figure 2:**
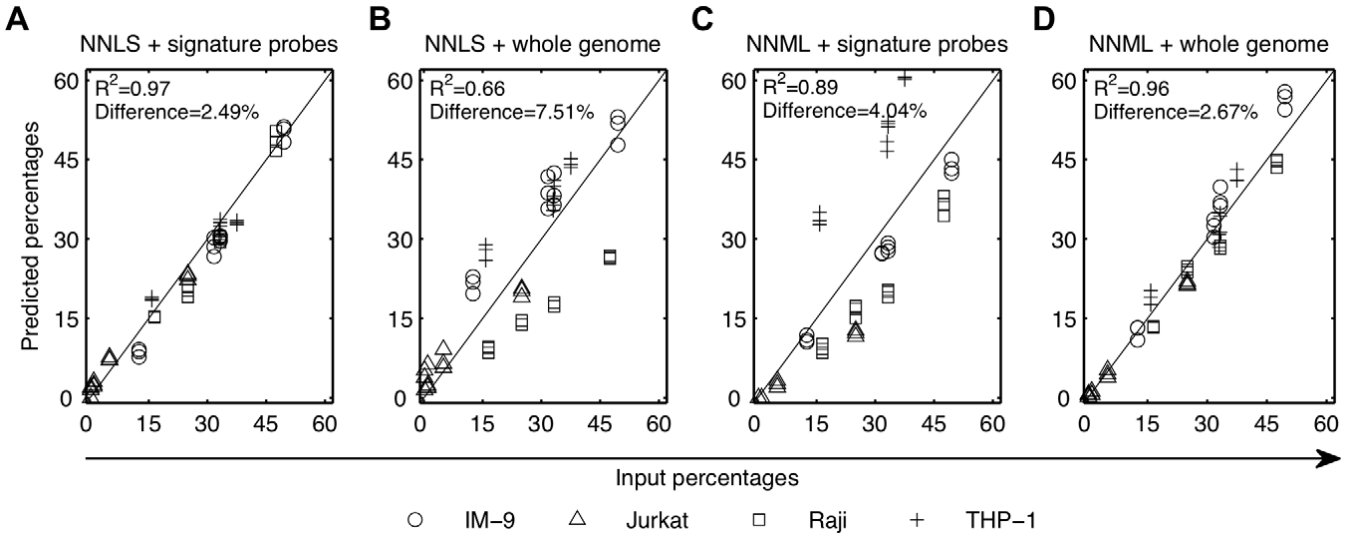
NNML recovers known compositions of immune cell line mixtures. Microarray data of IM-9 (○), Jurkat (▵), Raji (□), THP-1 (+), and the mixtures of these four cell lines in known proportions were obtained from Abbas et al. (2009). Proportions of each cell line were predicted using (A) NNLS with cell line signature probes (reproduced from Abbas et al. (2009)), (B) NNLS without cell line signature probe, (C) NNML with cell line signature probes, and (D) NNLS without cell line signature probes. Model predictions were compared with the input proportions used to create the mixtures. Cell line signature probes were obtained from Abbas et al. (2009).

To compare deconvolution accuracy (R^2^) and averaged absolute differences between the linear regression and LDA-based probabilistic models, we used archival gene expression data of heterogeneous samples created by mixing RNA samples of Raji, Jurkat, IM-9 and THP-1 cell lines in known proportions [3]. Compositions of the RNA mixtures were deconvolved using NNLS and NNML with gene expression profiles of 54,613 Affymetrix probes. The model predicted cell proportions were benchmarked against the results from [3] (Figure 2A), which were obtained using a NNLS model with an optimal number of 275 signature probes per cell line that were selected to maximize transcriptional distinction between the cell lines.

The deconvolution accuracy achieved by NNML using the 54,613 probes (Figure 2D) was only 0.01 lower than that achieved by NNLS using the optimized signature probes (Figure 2A), and the averaged absolute difference of NNML was 0.18% higher. For NNML using the optimized probes, the deconvolution accuracy (Figure 2C) was 0.08 lower than that of NNLS (Figure 2A), and the averaged absolute difference was 1.55% higher. In contrast, deconvolution accuracy of NNLS using all the probes (Figure 2B) was 0.25 lower than that of NNLS using the optimized probes, and the averaged absolute difference was 5.02% higher.

In this cell line analysis, the mixed profiles were derived from mixtures of RNA samples of 4 cell lines; there was no opportunity for microenvironmental or developmental factors to influence the gene expression of the reference and the constituent populations. Our analysis establishes a baseline that the LDA-based probabilistic model eliminates the need for cell line signature probes while performing deconvolution as accurately as the linear regression model with carefully optimized cell line signature probes, when the reference profiles match the constituent profiles of heterogeneous samples (Figures S1, S2, S3 in Text S1).

### Homogeneous populations with identical phenotypes exhibit varied transcriptional programs under varied environmental conditions

**Figure 3:**
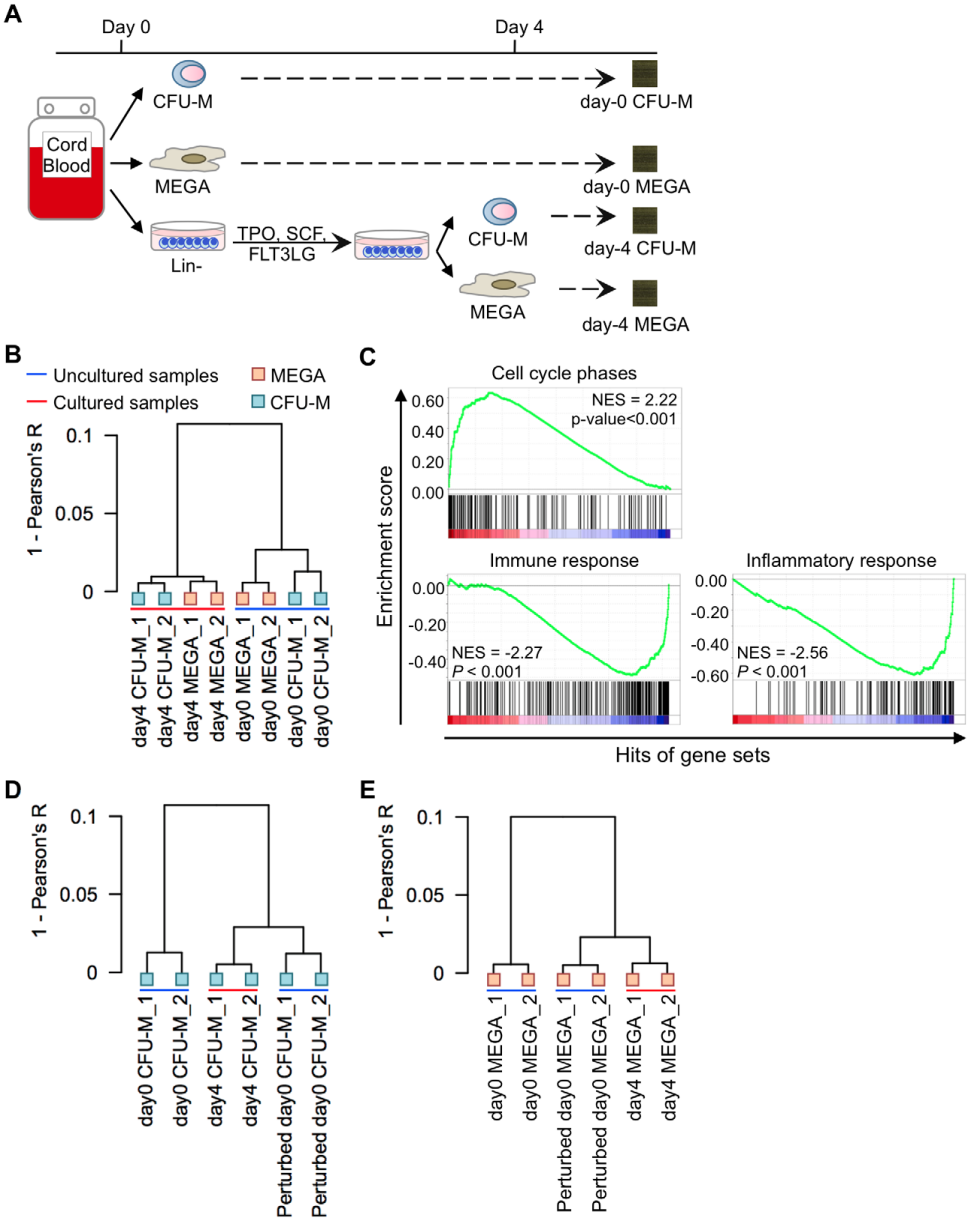
PERT captures cell culture effects. (A) Experimental setup for profiling genome-wide transcriptome expression of uncultured (day-0) and culture-derived (day-4) colony forming unit-monocytes (CFU-M) and megakaryocytes (MEGA). Lin-: lineage-depleted cells; TPO: thrombopoietin; SCF: stem cell factor; FLT3LG: fms-related tyrosine kinase 3 ligand. (B) Pearson's correlation comparison between day-0 and day-4 samples. (C) Plots of Gene Ontology enrichment analysis showing the enrichment scores of cell cycle phase genes, immune response genes, and inflammatory response genes by day-4 samples compared with day-0 samples. NES denotes the normalized enrichment score. P-values (*P*) were calculated using the hypergeometric test. (D) Pearson's correlation comparison between day-0 CFU-M, day-4 CFU-M, and perturbed day-0 CFU-M (or model predicted day-4 CFU-M) gene expression profiles. (E) Pearson's correlation comparison between day-0 megakaryocyte, day-4 megakaryocyte, and perturbed day-0 megakaryocyte (or model predicted day-4 megakaryocyte) gene expression profiles.

Analysis of blood progenitor cell surface antigens is a widely used surrogate for cellular functional properties, despite widespread recognition that this parameter is dynamic, especially on culture-derived cells [13]. Assuming that functional properties of a cell population are encoded by its transcriptional program, we hypothesized that cells from different microenvironmental and developmental conditions exhibit varied transcriptional programs despite their identical presentation of cell surface antigens. To validate this hypothesis, we compared genome-wide transcriptome profiles of uncultured and culture-derived blood mature cells and progenitor cells. The experimental protocol is shown in Figure 3A. In brief, megakaryocytes and colony forming unit-monocytes (CFU-M) were sorted from fresh (day-0) human umbilical cord blood. Enriched Lin- cells from the same umbilical cord blood samples were cultured as described in [14]. Megakaryocytes and CFU-M were harvested on day 4 using the same cell surface antigens and gating strategies as for day-0 samples (Figure S4 in Text S1). Gene expression profiles of the uncultured (day-0) and culture-derived (day-4) cells were obtained. As all the samples were prepared by following the same technical procedure, no batch removal analysis of gene expression data was performed. Figure 3B shows that robust multi-array average (RMA) [15] normalized gene expression profiles of the day-0 and day-4 samples segregated into “uncultured” and “cultured” clusters based on their Pearson's correlation coefficients, instead of “megakaryocyte” and “CFU-M” clusters as would be expected from a functional perspective. Gene set enrichment analysis (GSEA) [16] suggested that genes up-regulated in day-4 samples compared to day-0 samples were enriched in cell cycle related processes, and those down-regulated were enriched in immune and inflammatory responses (Figure 3C, Table S1). We anticipated that a “cell culture effect” had caused uncultured and culture-derived cells expressing the same lineage-associated surface antigens to exhibit different transcriptional programs.

We then explored if PERT could capture and account for the cell culture effect. The model was applied to day-0 and day-4 megakaryocytes (or CFU-M) to estimate a genome-wide multiplicative perturbation vector, ***ρ***, to capture gene-specific cell culture effects (Table S2). GSEA was applied to the genes whose expression levels had been perturbed by more than 2-fold (*ρ_g_*<0.5 or *ρ_g_*>2) when comparing day-4 megakaryocytes with day-0 megakaryocytes, and day-4 CFU-M with day-0 CFU-M. We found that the GSEA results for megakaryocytes (Table S3) and CFU-M (Table S4) were similar. Overall, the day-4 samples exhibited higher expression of cell cycle, cell division, DNA and RNA metabolic processes and cell component assembly related genes (Conditional hypergeometric test [17], *P*<0.01), and the day-4 samples exhibited a decrease in expression of immune system related genes (Conditional hypergeometric test [17], *P*<0.01). These results were consistent with the results shown in Figure 3C and Table S1, suggesting that PERT had captured the cell culture effects. The ***ρ*** vector from comparing day-4 with day-0 megakaryocytes (or from comparing day-4 with day-0 CFU-M) was then applied to the gene expression profiles of day-0 CFU-M (or day-0 megakaryocytes) to obtain perturbed gene expression profiles of day-0 CFU-M (or day-0 megakaryocyte). As shown in Figure 3D (or 3E), the perturbed gene expression profiles of day-0 CFU-M (or day-0 megakaryocyte) exhibited a stronger Pearson's correlation with that of day-4 CFU-M (or day-4 megakaryocyte) than the original gene expression profiles of day-0 CFU-M (or day-0 megakaryocyte), confirming the success of PERT in estimating systematic effect of cell culture on reference profiles (Figures S5 and S6 in Text S1).

### PERT recovers constituent proportions of uncultured human umbilical cord blood samples

**Figure 4:**
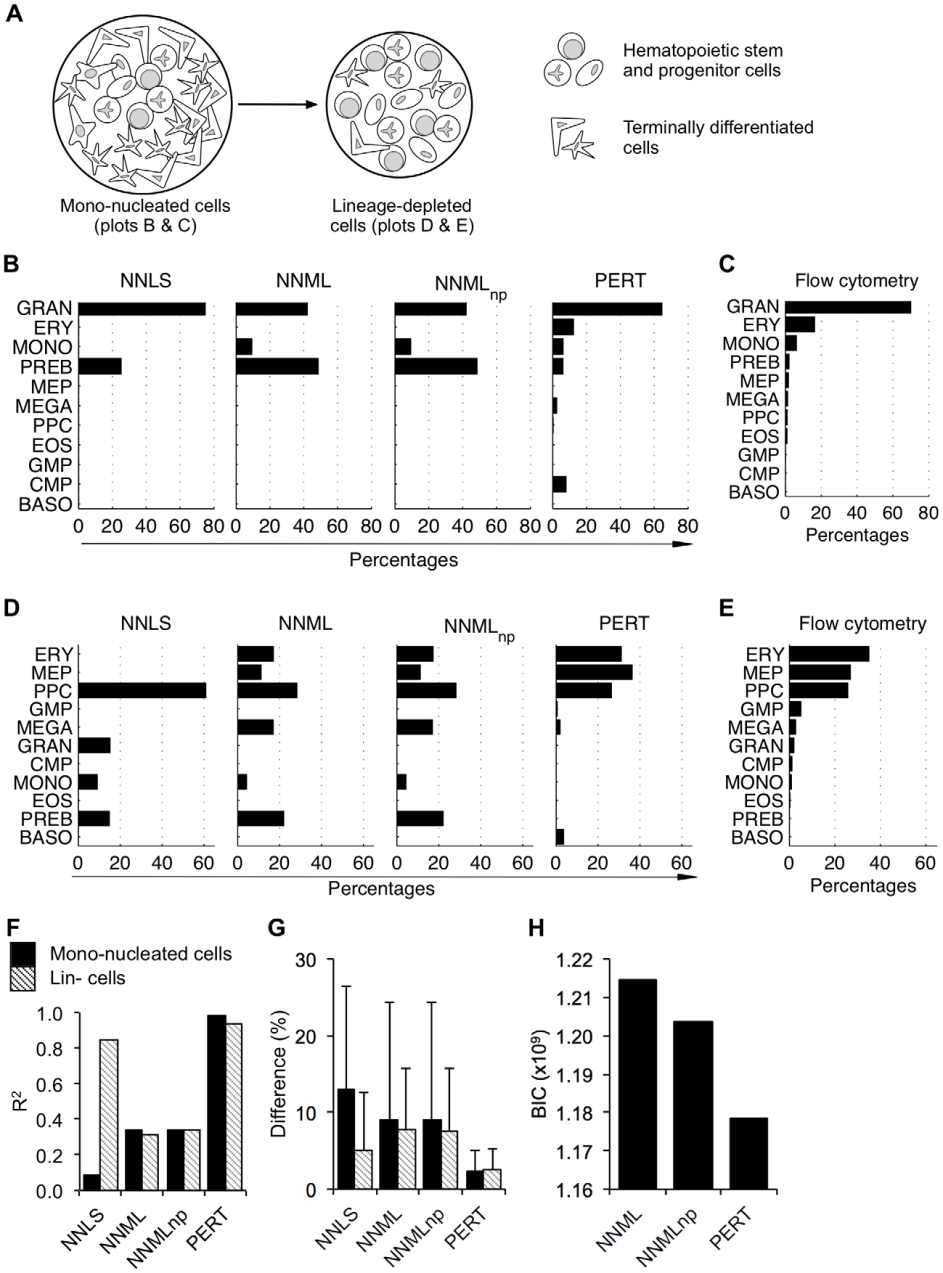
PERT recovers compositions of uncultured human cord blood mono-nucleated and lineage-depleted (Lin-) cells. (A) Schematic compositions of mono-nucleated cell samples and Lin- cell samples. (B) Model predicted proportions of 11 homogeneous blood cell lineages, namely granulocytes (GRAN), erythrocytes (ERY), monocytes (MONO), precursor B cells (PREB), megakaryocyte-erythrocyte progenitors (MEP), megakaryocytes (MEGA), primitive progenitor cells (PPC), eosinophils (EOS), granulocyte-monocyte progenitors (GMP), common myeloid progenitors (CMP), and basophils (BASO) in uncultured human mono-nucleated cord blood cell samples. (C) Flow cytometry measured proportions of the 11 blood cell lineages in the uncultured human mono-nucleated cord blood cell samples shown in (B). (D) Model predicted proportions in uncultured human Lin- cord blood cell samples. (E) Flow cytometry measured proportions in the uncultured human Lin- cord blood cell samples shown in (D). (F) R^2^ calculated from the Pearson's correlation coefficients between the model predicted cell proportions and the ones assigned by flow cytometry. See Table 2 for the associated t-statistics and P-values. (G) Averaged absolute differences of model predicted cell proportions. Error bars show standard deviations of the absolute differences between model predicted and flow cytometry assigned proportions of the 11 blood cell lineages. (H) The Bayesian information criterion (BIC) calculated from the parameters in Table 1.

Having established that expression deconvolution was accurate for samples where all constituent populations were known and that PERT could capture systematic transcriptional variations between uncultured populations and the cultured versions of those populations, we then used the four models — NNLS, NNML, NNML_np_ and PERT — to deconvolve uncultured human mono-nucleated and Lin- umbilical cord blood samples (Figure 4A) where compositions are not pre-specified.

Mixed profiles of mono-nucleated cells enriched from fresh human umbilical cord blood were first deconvolved to estimate the proportions of 11 developmentally and functionally distinct blood populations (Table S5 and Text S1) using their reference profiles from [18]. As expected, because the two sets of samples were obtained by different labs, batch effects between the mixed profiles and the reference profiles were observed, and these were removed using the supervised normalization of microarray (SNM) method [19]. We benchmarked the model predicted cell proportions (Figure 4B and Table S6) against those measured by flow cytometry (Figure 4C and Table S6) using the same cell surface antigens originally used to recover the reference populations in [18]. The same analysis was performed for fresh human umbilical cord blood-derived Lin- cell samples (Figures 4D and 4E, and Table S6), which are known to have different compositions from mono-nucleated cell samples. The gene expression profile ***γ*** of the new reference population from NNML_np_ and the perturbation vector ***ρ*** from PERT are given in Table S7. Results of GSEA for genes whose perturbation factor *ρ_g_* is <0.5 or >2 are in Table S8.

Notably, the deconvolved proportions of uncultured mono-nucleated cell samples and Lin- cell samples using NNML and that of NNML_np_ were not substantially different (*P* = 2.43×10^−1^) (Figures 4F and 4G). For mono-nucleated cell samples, there was a large improvement in the deconvolution performance of PERT compared to the other three models in terms of both the deconvolution accuracy R^2^ and the averaged absolute differences (Figures 4F and 4G). However, for Lin- cell samples, while the deconvolution accuracy R^2^ of NNLS and PERT were both high, the absolute differences of PERT were significantly lower than that of NNLS (*P* = 5.00×10^−3^). The Bayesian information criterion (BIC) indicated preferential applicability of PERT in deconvolving these uncultured heterogeneous samples (Table 1 and Figure 4H).

**Table 1:** Parameters of NNML, NNML_np_ and PERT for the Bayesian information criterion (BIC) calculations shown in Figure 4H and Figure 5F.

	Uncultured mono-nucleated and lineage-depleted cell samples			Culture-derived (day-4) lineage-depleted cell samples		
	NNML	NNML_np_	PERT	NNML	NNML_np_	PERT
N_reference_	118	118	118	118	118	118
N_heterogenous_	4	4	4	4	4	4
N_probes_	-	22215	22215	-	22215	22215
θ	468	468	468	468	468	468
ω	0	4	0	0	4	
κ	0	1	1	0	1	1
α	0	4	4	0	4	4
β	0	22214	0	0	22214	0
ρ	0	0	22215	0	0	22215
N_parameters_	468	45039	45036	468	45039	45036
N_observations_	68133988	68133988	68133988	45933224	45933224	45933224
In( 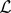 )	−6.07E+08	−6.02E+08	−5.89E+08	−4.13E+08	−4.00E+08	−3.91E+08
BIC	1.21E+09	1.20E+09	1.18E+09	8.26E+08	8.00E+08	7.83E+08

**Table 2:** Associated statistics for the Pearson's correlation analysis between the model predicted and flow cytometry assigned cell proportions for uncultured mono-nucleated and lineage-depleted cell samples enriched from fresh human umbilical cord blood.

	Mono-nucleated cells samples			Lineage-depleted cells samples		
Models	R	t-stats	P-value	R	t-stats	P-value
NNLS	0.29	0.91	0.39	0.92	7.04	0.00
NNML	0.58	2.14	0.06	0.56	2.03	0.07
NNML_np_	0.58	2.14	0.06	0.58	2.14	0.06
PERT	0.99	21.05	0.00	0.97	11.97	0.00

R: Pearson's correlation coefficients.

This analysis indicates that PERT recovered cell proportions of 11 reference populations with averaged absolute differences as low as 2%. In addition, PERT only required two biological samples of mono-nucleated cells and Lin- cells, and 4 to 10 biological profiles of individual reference populations, whereas flow cytometry required preparation of 41 aliquot samples (including controls) to measure the proportions of the same constituent populations as the deconvolution analysis in one mono-nucleated or Lin- cell sample.

### PERT recovers constituent proportions of culture-derived human blood samples

**Figure 5:**
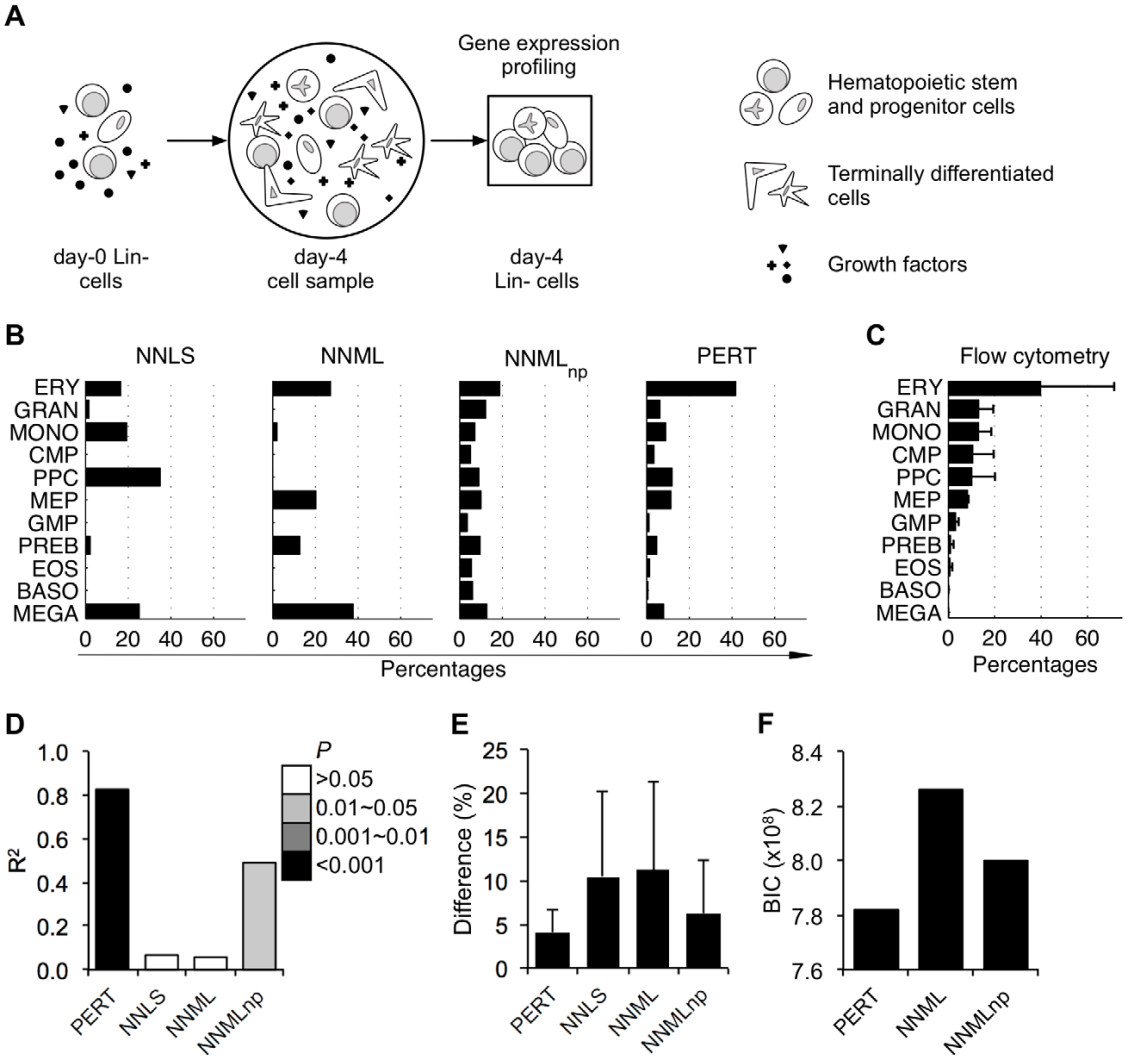
PERT recovers compositions of culture-derived lineage-depleted (Lin-) human blood cells. (A) Schematic of experiment setup. (B) Model predicted cell proportions of 11 blood cell lineages (defined in Figure 4) in day-4 Lin- human blood cell samples. (C) Flow cytometry assigned averaged cell proportions (N = 3) in the day-4 Lin- human blood cell samples shown in (B). (D) R^2^ calculated from the Pearson's correlation coefficients between the model predicted cell proportions and the ones assigned by flow cytometry. (E) Averaged absolute differences of model predicted cell proportions. Error bars show standard deviations of the absolute differences of the 11 blood cell lineages. (F) The Bayesian information criterion (BIC) calculated from the parameters in Table 1.

Having established that PERT could capture culture-associated changes in gene expression in relatively pure populations (analysis of day-4 versus day-0 megakaryocytes and CFU-M) and microenvironment-associated changes in heterogeneous samples (analysis of uncultured mono-nucleated and Lin- cell samples), we next applied the model to analyze culture-derived heterogeneous samples from a hematopoietic stem and progenitor cell (HSPC) expansion culture. The experimental setup is described in detail elsewhere [20]. In brief, human umbilical cord blood Lin- cells were seeded in a suspension culture that had been optimized for HSPC expansion. After 4 days, Lin- cells were harvested, and then their genome-wide transcriptome expression was profiled (Figure 5A).

Proportions of the 11 blood cell lineages [18] were deconvolved (Table S5 and Figure S8 in Text S1). Model predictions (Figure 5B and Table S6) were validated by the cell proportions assigned by flow cytometry (Figure 5C and Table S6). The deconvolution accuracy R^2^ of PERT was significantly higher than that of the other models (Figure 5D), and the averaged absolute differences of PERT were lower as assessed by the Wilcoxon signed rank test (*P* for PERT versus NNLS, PERT versus NNML, and PERT versus NNML_np_ were 9.00×10^−3^, 1.00×10^−3^ and 1.39×10^−1^, respectively) (Figure 5E). In addition, the BIC (Table 1 and Figure 5F) indicates preferential applicability of PERT in this case. Intriguingly, compared with the results for uncultured samples for which deconvolution accuracy R^2^ and averaged absolute differences of NNML and NNML_np_ were not significantly different, the predictions of NNML_np_ were much more correlated (R^2^ = 0.49 versus R^2^ = 0.06) with the cell proportions in the culture-derived samples than the NNML model, although the averaged absolute differences of the two models were similar.

GSEA was performed for genes identified by PERT as being perturbed in the mixed profiles by more than 2-fold over the reference profiles (Table S9). Cultured-derived Lin- cells were found to upregulate genes enriched in cell cycle, metabolic and catabolic processes, and biosynthetic processes (Conditional hypergeometric test [17], *P*<0.01) (Table S10).

Collectively, this analysis showed that PERT recovered cell proportions of culture-derived heterogeneous samples using the gene expression profiles of uncultured reference populations. PERT analysis revealed that transcriptome differences between uncultured and culture-derived cells of the same phenotypic identity were attributable to the increased expression of cell cycle process related genes by the culture-derived cells.

## Discussion

We have demonstrated that the transcriptional variations due to microenvironmental and developmental differences could not be addressed using existing batch effect models in gene expression deconvolution. We have introduced PERT, a new deconvolution method that allows for transcriptional variations between reference populations and constituent populations in heterogeneous samples of interest.

Transcriptional programs of human cells fluctuate with circadian rhythms and vary among individuals [21]. Furthermore, procedures of blood collection, cell isolation and RNA extraction affect the expression of specific genes [22]. As reference profiles and mixed profiles are often collected by different labs, available reference profiles may not accurately represent the corresponding constituent populations composing the mixed profiles, even though they have the same cell surface markers. Gene expression differences between the reference profiles and the constituent profiles cannot be accounted for by the existing batch effect models because they assume that the reference and the constituent populations are the same, except for technical differences in data collection.

Differences in performance of the four models for culture-derived samples may be explained by one of several factors that can complicate deconvolution. First, progenitor cells in culture can differentiate and give arise to intermediate cell types or populations that are not included in the reference populations. This could explain why NNML_np_ captured seven times more compositional variation than NNML when they were used on culture-derived Lin- cells, but the two models produced similar results when they were used on uncultured samples. Second, culture-derived heterogeneous samples and reference samples which were directly isolated from patient samples had been exposed to different environments. Cell extrinsic factors cause genome-wide transcriptional variations [23] between the reference and constituent profiles. We found that these variations were not easily captured by modeling the presence of a new population in heterogeneous samples as is done by NNML_np_. In contrast, modeling these variations by a systematic genome-wide perturbation to the reference profiles as done by PERT was more successful.

We anticipate that the improved performance of PERT in deconvolving heterogeneous samples over the other tested models herein is attributed to its more flexible and appropriate model assumptions. First, accumulating evidence has indicated the association between cell phenotypes and molecular networks consists of relatively small numbers of genes out of the whole genome [18]. Although components of cell phenotype-associated molecular networks can be used as cell signature genes for NNLS deconvolution, identification of those components is challenging, especially for a large number of cell types within the hematopoietic system because mature hematopoietic cells are generated from hematopoietic stem and progenitor cells through an amplifying differentiation hierarchy and the transcriptional profiles that distinguish different but related cell types is still very much an area of active investigation [18], [24]. Second, definition of cell type signature genes is technically subjective. Third, although NNML eliminates the need to identify cell type signature genes, the model assumes that each constituent population is represented by one or more reference populations, and that the reference profiles are accurate estimates of the profiles of the constituent populations. However, reference profiles are rarely accurate estimates of the constituent profiles in practice due to the effects of environmental factors, technical factors and cell-cell interactions on gene expression that often occur in cell culture. While NNML_np_ can help address the problem of an incomplete reference profile set, it cannot account for systematic variations in reference and constituent profiles. PERT is the first step towards addressing these transcriptional variations due to culture conditions. A future development of PERT could be to estimate a perturbation factor for each reference population to represent cell type specific culture effect, as opposed to the shared perturbation factor used here. Such a model would be similar to an expression deconvolution model in which both the reference populations and their proportions were unknown with a strong prior to guide the deconvolution and ensure identifiability. We suspect that such model would require more data to fit.

Here we demonstrated success in applying *in silico* techniques to deconvolve compositions of heterogeneous samples using reference profiles collected under different conditions. As a large amount of resource and energy is required to generate a comprehensive data set of reference profiles, the ability to use available reference profiles to decompose heterogeneous samples potentially collected from different environmental conditions should dramatically extend the utility of archival gene expression datasets. Selection of a proper deconvolution model can be challenging in the situation where the nature or content of mixed samples is uncertain. In this work, we explored R^2^, averaged absolute differences, and BIC as a means to select between NNLS, NNML, NNML_np_ and PERT. Intriguingly, we found that PERT performed as well as, or better than the other models in all tested cases. The model has allowed us to recapitulate flow cytometry estimated cellular compositions of heterogeneous samples in a more efficient, unbiased manner. Our results demonstrated the importance of including prior knowledge of biological systems (e.g., existence of new cell populations, transcriptional variations between reference and constituent populations) to achieve excellent deconvolution accuracy. We anticipate that PERT is not only relevant to the hematopoietic system, but is applicable to any heterogeneous biological system given prior knowledge about the gene expression profiles of reference populations.

## Materials and Methods

### Non-negative least squares model (NNLS) formulation

In the following model description, variables are in italics, constants are in uppercase, and vectors are in bold. All deconvolution models herein make several common assumptions. They assume that the input consists of two sets of expression profiles. One set consists of D heterogeneous profiles corresponding to the gene expression profiles of D heterogeneous samples, where ***x*** *_d_* is a vector of length G and *x_d,g_* is the discretized total intensity measurement for gene *g* in sample *d*. The other set consists of K reference profiles corresponding to the gene expression profiles of K reference cell populations, where ***v*** *_k_* is a vector of length G and *v_k,g_* is the total intensity measurement for gene *g* in reference population *k*.

The standard formulation for deconvolution is to model each heterogeneous profile ***x*** *_d_* as a linear combination of measurements of the reference populations, ***v*** *_k_*, weighted by mixture proportions ***θ*** *_d_*:
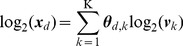
(1)


We used log_2_ transformed gene expression data and the nnls() function from the nnls package (version 1.4) of R to estimate the optimal non-negative values of *θ_d,k_* as previously described [9]. We then re-scaled the values *θ_d,k_* such that Σ_k_ *θ_d,k_* = 1 as done in [3].

There are several limitations with the NNLS model that we aimed to address in this work. First, NNLS requires cell type signature genes. However, identifying cell type-specific signature genes for different but related reference populations is challenging (Text S1). Second, as shown below, probabilistic representations of deconvolution can be naturally extended to estimate the profile of an additional (unknown) reference population, or to explicitly model the effects of cell culture on the gene expression profiles of cells.

### Non-negative maximum likelihood model (NNML) formulation

NNML is a probabilistic alternative to NNLS, which uses a different noise model that is less sensitive to the selection of cell type signature genes and also provides a basis upon which to address the estimation of an unknown reference population (NNML_np_) or cell culture effects (PERT). NNML treats heterogeneous expression profiles as digital measurements of gene abundances in a sample: that is, *x_d,g_* represents a count of the number of times that gene *g* was found in sample *d* as measured in arbitrary units of intensity or read density. In other words, there are *x_d,g_* observations of a unit of intensity. We model each of those *x_d,g_* observations as coming from exactly one constituent population; *x_d,g_* is therefore the sum of contributions from each of the constituent cell populations present in the heterogeneous sample, and N*_d_* = Σ_g_ *x_d,g_* is the total number of observations for sample *d*. In this work, the units are selected so that N*_d_* is on the order of 10^7^. The goal of deconvolution is to estimate *θ_d,k_*, the fraction of all observations in sample *d* attributable to reference population *k*, by identifying from which reference population each observation originates.

In order to infer from which reference population each observation originates, we expand each heterogeneous expression profile from the compact vector ***x*** *_d_* into an alternative vector ***t*** *_d_* of length N*_d_*, where *t_d,n_* ∈ {1,…,G} represents the *n* ^th^ observation from sample *d*. Note that the vectors ***t*** *_d_* and ***x*** *_d_* store the same information because Σ_n_[*t_d,n_* = g] = *x_d,g_*, where [*t_d,n_* = g] is the indictor function that is 1 if *t_d,n_* = g, and otherwise 0. Representing heterogeneous profile *d* using the vector ***t*** *_d_* allows us to simplify the deconvolution problem to inferring a vector ***z*** *_d_* of length N*_d_*, where *z_d,n_* = k indicates that the observation *t_d,n_* originated from reference population *k*. Inference of all *z_d,n_* variables allows straightforward estimation of *θ_d,k_*; we can set *θ_d,k_* = Σ_n_[*z_d,n_* = k]/N*_d_*.

Also, because NNML treats heterogeneous expression profiles *t_d,n_* as digital measurements, it is natural to treat each observation *t_d,n_* as a draw from a discrete distribution, whose parameters characterize the expression profile of the sample *d*. We first converted each of the reference expression profiles ***v*** *_k_* into parameters of a discrete distribution ***β*** *_k_*, where *β_k,g_* = *v_k,g_*/N*_k_* and N*_k_* = Σ_g_ *v_k,g_*. For each observation *t_d,n_* in heterogeneous sample *d*, conditioned on the knowledge of which constituent population it is from (i.e. knowledge of *z_d,n_*), the likelihood of observing the specific gene *t_d,n_* is defined by the appropriate reference distribution 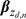.

NNML makes two limiting assumptions. First, it assumes that all constituent populations of each heterogeneous sample are represented by at least one discrete distribution ***β*** *_k_* from the provided reference profiles. Second, it assumes that each reference profile ***β*** *_k_* faithfully recapitulates the gene expression pattern of the corresponding cell type *k* in each heterogeneous sample. Under these assumptions, NNML estimates ***θ*** *_d_* by maximizing the following complete log likelihood function using conjugate gradient descent until convergence of the likelihood function:
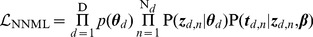
(2)

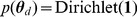
(3)

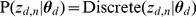
(4)

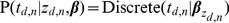
(5)


The initial states of the hidden variables ***θ*** *_d_* are all set to 1/K before optimization. See Program S2 for the NNML program. NNML deconvolution was performed on linear, untransformed gene expression data.

### Non-negative maximum likelihood new population model (NNML_np_) formulation

NNML_np_ is an extension of NNML. This model relaxes NNML's assumption that all constituent populations in each heterogeneous sample are represented in the provided reference sets ***β*** *_k_*. Namely, NNML_np_ assumes that there exists a single cell population ***γ*** that is not in the reference set ***β*** *_k_* but that is present in at least one of the heterogeneous samples. NNML_np_ is a slightly modified version of the ISOLATE [5] model that we reported previously. In order to prevent overfitting in the estimation of ***γ***, we place a prior over ***γ*** such that ***γ*** is drawn from a Dirichlet distribution centred on a convex combination of the existing reference populations ***β*** *_k_* because we assume that, all else being equal, the new population will be related to the existing reference populations. The convex weights ***ω***, as well as the strength of the prior *κ*, are estimated from the data. Finally, NNML_np_ also puts a Dirichlet prior over each variable ***θ*** *_d_* to prevent overfitting: that prior has mean ***α*** that is also estimated. Estimating the hidden variables and parameters (***γ***, ***ω***, *κ*, ***α*** and ***θ*** *_d_*) are optimized by (block) coordinate descent; the complete log likelihood function is cyclically optimized with respect to each set of hidden variables and parameters using conjugate gradient descent, until convergence of the likelihood function. The complete likelihood function is as follows (variables ***θ*** *_d_*, *t_d,n_*, *z_d,n_*, and ***β*** *_k_* have the same meaning as for NNML):
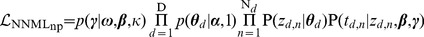
(6)

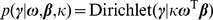
(7)

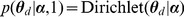
(8)

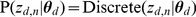
(9)

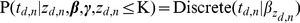
(10)

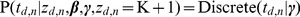
(11)


Initialization of model parameters is described in the Text S2. The major difference between NNML_np_ and ISOLATE is that the Dirichlet prior on the new population (eq. 7) in NNML_np_ is replaced with a product of Gamma priors in ISOLATE. See Program S2 for the NNML_np_ program. NNML_np_ deconvolution was performed on linear, untransformed gene expression data.

### Perturbation model (PERT) formulation

In contrast to NNML_np_, PERT extends NNML by relaxing its other main assumption, namely, that the provided reference distributions ***β*** *_k_* faithfully represent the expression patterns of the actual constituent cell populations in each heterogeneous sample. PERT defines new constituent profiles ***γ***_1_ through ***γ***_K_, where ***γ*** *_k_* is based on the reference profile ***β*** *_k_* that has been adjusted for systematic differences due to cell culture effects, for example. These systematic changes in gene expression are assumed to act equally across all constituent cell populations, and are defined by multiplicative perturbation factors *ρ_g_*. PERT uses a prior distribution over *ρ_g_*, with a mean of one and strength of *κ*, to regularize *ρ_g_* such that it introduces as few deviations as possible. Similar to NNML_np_, we introduce a prior over ***θ*** *_d_* for regularization, where the mean of that prior, ***α***, is also estimated. Estimating hidden variables and parameters (*ρ_g_*, *κ*, ***α*** and ***θ*** *_d_*) is done by cyclically optimizing the complete log likelihood function with respect to each hidden variable and parameter using conjugate gradient descent, until convergence of the likelihood function. The likelihood function is as follows (variables ***θ*** *_d_*, *t_d,n_*, *z_d,n_*, and ***β*** *_k_* have the same meaning as for NNML):
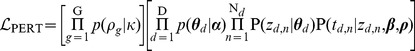
(12)

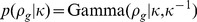
(13)

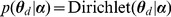
(14)

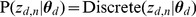
(15)

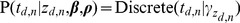
(16)

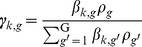
(17)


Initialization of model parameters is described in the Text S2. See Program S3 for the PERT program. PERT deconvolution was performed on linear, untransformed gene expression data.

### Model implementation

NNML, NNML_np_ and PERT were implemented in Matlab, and the programs were used to obtain the results herein. The Matlab programs were converted into Octave to allow them to be used with free software. The programs are found in the supporting information (See instructions in Text S2).

### Microarray preparation for mono-nucleated cell and lineage-depleted cell samples

Samples of human umbilical cord blood were obtained from Mount Sinai Hospital (Toronto, ON, Canada) and processed in accordance to guidelines approved by the University of Toronto. Mono-nucleated cells were obtained by lysing the erythrocytes. Lineage-depleted (Lin-) cells were isolated from mono-nucleated cells using the EasySep system (Stemcell Technologies, Vancouver, BC, Canada) according to the manufacture's protocol.

Genome-wide expression of mono-nucleated cells and Lin- cells were profiled by isolating total RNA using Rneasy Mini kits (Qiagen). RNA quality was tested on both NanoDrop (ND-1000) and BioAnalyzer machines. cDNA samples were prepared using Nugen IVT kit, and split into 2 technical replicates. Hybridization was performed using Affymetrix Gene Chip HG-U133A2.0 arrays on the Affymetrix Gene Chip Scanner 3000 machine.

### Microarray preparation for CFU-M and megakaryocytes

CD34^−^CD33^+^CD13^+^ colony forming unit-monocytes (CFU-M) and CD34^−^CD41^+^CD61^+^CD45^−^ megakaryocytes were sorted from pooled fresh human umbilical cord blood samples on BD FACS Aria (CD34: PE; CD33: APC; CD13: PERCP; CD41: PE; CD61: FITC; CD45: APC. All antibodies were purchased from BD BioScience). Lin- cells were cultured as described in [14]. On day 4, CFU-M and megakaryocytes were sorted. Total RNA of the four samples was isolated using RNeasy Micro kit (Qiagen). RNA quality was tested on both NanoDrop (ND-1000) and BioAnalyzer machines. cDNA samples were prepared using Ambion IVT kit. Hybridization was performed using Affymetrix HG-U133Plus2 arrays on the Affymetrix Gene Chip Scanner 3000 machine. Data of two biological replicates were collected.

### Flow cytometry

Compositions of mono-nucleated cells and Lin- cells were analyzed by flow cytometry on either BD FACS Canto Flow Cytometer or BD LSRFortessa. Data analysis was performed with BD FACSDiva Software version 5.0.1.

### Downloaded microarray data sets

Normalized gene expression data (Affymetrix Gene Chip HG-U133Plus2) of IM-9, Jurkat, Raji, THP-1 cell lines, and mixtures of the four cell lines were downloaded from the Gene Expression Omnibus (GSE11103; downloaded on 23^rd^ August 2012). Affymetrix CEL files (Affymetrix Gene Chip HG_U133AAofAv2) of 21 human umbilical cord blood-derived pure populations (Table S5) were obtained from the authors of [18] (GSE24759). Affymetrix CEL files (Affymetrix Gene Chip HG-U133Plus2) of day-4 Lin- cells were obtained from the authors of [20] (GSE16589).

### Microarray pre-processing and batch effect removal

Microarray data were analyzed in BioConductor using the affy package. For the analysis of CFU-M and megakaryocyte profiles, RMA [15] background adjusted, normalized profiles, without batch removal, were used because all the samples for this analysis were processed under the same technical setup. The processed data of CFU-M and megakaryocyte samples are found in Table S11. For the deconvolution studies of uncultured and culture-derived samples, RMA [15] background adjusted, non-normalized reference and mixed profiles were post-processed by the supervised normalization of microarray (SNM) method [19] in order to normalize data while removing the batch effects between the two datasets. The processed data of uncultured and culture-derived samples are found in Table S12 and Table S13, respectively.

### Hierarchical clustering

Hierarchical clustering shown in Figure 3 was obtained from log_2_ gene expression values using an average agglomeration method with a distance matrix of (1 - Pearson's correlation coefficients).

### Gene set enrichment analysis

GSEA was either done using the GSEA program (v2.0) from the GSEA website using gene sets c5.all.v3.0.orig.gmt (downloaded on Jan 23, 2012), or using the GSEAStat (v2.20.0) and GSEABase (v1.16.0) packages with the generic GOslim gene sets (download from the GSEA website on Jan 21, 2012) in the BioConductor.

### Statistics analysis

Unless otherwise stated, all P-values were calculated using the Wilcoxon signed rank test in R. Association test of Pearson's correlation was done in R using the cor.test() function.

### Accession codes

Gene Expression Omnibus, GSE40831.

## Supporting Information

Program S1 **Octave program for NNML.**
(ZIP)

Program S2 **Octave program for NNML_np_.**
(ZIP)

Program S3 **Octave program for PERT.**
(ZIP)

Table S1 **Gene ontology difference between culture-derived and uncultured blood cell samples.** Gene set enrichment analysis was performed for pooled day-4 CFU-M and day-4 megakaryocyte profiles and pooled day-0 CFU-M and day-0 megakaryocyte profiles.
(XLS)

Table S2 **Gene-specific perturbation factors obtained from comparing culture-derived samples to uncultured samples.** (A) Perturbation vectors ***ρ*** from comparing gene expression profiles of day-0 megakaryocytes to that of day-4 megakaryocytes. (B) Perturbation vectors ***ρ*** from comparing gene expression profiles of day-0 CFU-M to that of day-4 CFU-M.
(XLS)

Table S3 **Enriched biological processes of the perturbed genes when comparing culture-derived to uncultured megakaryocytes.** Gene expression profiles of day-4 megakaryocyte were compared to that of day-0 megakaryocytes using PERT. Gene set enrichment analysis was performed for Affymetrix probes that exhibited 2-fold perturbation (*ρ_g_*<0.5 or *ρ_g_*>2). The enriched gene sets (*P*<0.01) are tabulated.
(XLS)

Table S4 **Enriched biological processes of the perturbed genes when comparing culture-derived to uncultured CFU-M.** Gene expression profiles of day-4 CFU-M were compared to that of day-0 CFU-M using PERT. Gene set enrichment analysis was performed for Affymetrix probes that exhibited 2-fold perturbation (*ρ_g_*<0.5 or *ρ_g_*>2). The enriched gene sets (*P*<0.01) are tabulated.
(XLS)

Table S5 **Reference populations for decomposing human cord blood samples.**
(XLS)

Table S6 **Comparison between flow cytometry-assigned and model-predicted cell compositions of different mixed samples.** (A) Mono-nucleated cells enriched from fresh human umbilical cord blood. (B) Lineage-depleted cells enriched from fresh human umbilical cord blood. (C) Lineage-depleted cells enriched from the 4^th^ day of hematopoietic stem and progenitor cell expansion culture.
(XLS)

Table S7 **NNML_np_ and PERT analysis for fresh human umbilical cord blood samples.** Gene expression profiles of mono-nucleated and lineage-depleted cell samples enriched from fresh human umbilical cord blood were analyzed by NNML_np_ and PERT. (A) The predicted gene expression profile ***γ*** of the new reference population obtained using NNML_np_. (B) The predicted perturbation vector ***ρ*** obtained using PERT.
(XLS)

Table S8 **Differences between biological properties of uncultured heterogeneous samples and that of reference populations.** Gene expression profiles of mono-nucleated and lineage-depleted cell samples enriched from fresh human umbilical cord blood were analyzed by PERT. Gene Ontology (GO) enrichment analysis was performed for Affymetrix probes that exhibited 2-fold up-regulation (*ρ_g_*>2) in the mixed profiles. Enriched GO terms (*P*<0.01) are tabulated.
(XLS)

Table S9 **NNML_np_ and PERT analysis for culture-derived human blood samples.** Gene expression profiles of cultured-derived lineage-depleted human blood cell samples were analyzed by NNML_np_ and PERT. (A) The predicted gene expression profile ***γ*** of the new reference population obtained using NNML_np_. (B) The predicted perturbation vector ***ρ*** obtained using PERT.
(XLS)

Table S10 **Differences between biological properties of culture-derived heterogeneous samples and reference populations.** Gene expression profiles of culture-derived lineage-depleted human blood cell samples were analyzed by PERT. Gene ontology (GO) enrichment analysis was performed for genes that exhibited 2-fold up-regulation (*ρ_g_*>2) in the mixed profiles. Enriched GO terms (*P*<0.01) are shown.
(XLS)

Table S11 **Processed gene expression profiles of CFU-M and megakaryocyte samples.**
(XLSX)

Table S12 **Gene expression profiles for deconvolving uncultured mono-nucleated and lineage-depleted cell samples.**
(XLS)

Table S13 **Gene expression profiles for deconvolving culture-derived lineage-depleted cell samples.**
(XLS)

Text S1 **Performance analysis of NNLS, NNML, NNML_np_ and PERT.**
(DOC)

Text S2 **Initialization and usage of NNML, NNML_np_ and PERT.**
(DOCX)
