# Supplementary material for: PERT: A Method for Expression Deconvolution of Human Blood Samples from Varied Microenvironmental and Developmental Conditions
Source: PLoS Comput Biol. 2012 Dec 20;8(12):e1002838. doi: 10.1371/journal.pcbi.1002838 (PMC3527275; doi:10.1371/journal.pcbi.1002838)
Supplement: Text S1 — Performance analysis of NNLS, NNML, NNMLnp and PERT. (DOC) [file pcbi.1002838.s017.doc]

**Text S1:**

**PERT: a method for expression deconvolution of human blood samples from varied microenvironmental and developmental conditions**

Wenlian Qiao, Gerald Quon, Elizabeth Csaszar, Mei Yu, Quaid Morris, Peter W. Zandstra

1. **Performance of NNML_np_ and PERT in decomposing physical mixtures of homogeneous samples**

We applied NNML_np_ and PERT to the data set consisting of reference profiles for four immune cell lines and expression profiles for mixtures of the cell lines in known proportions [1], a situation in which all constituent populations are represented in the reference profiles, and all expression profiles are collected under the same conditions. The R^2^ of NNML_np_ with the whole genome expression profiles was significantly lower than the other models (Figure S1A and Figure 2 in the main text). The deconvolved proportions for the new populations were >30%, much larger in comparison to the expected 0% (Figure S2A). Furthermore, the deconvolved new population profile was highly correlated to the reference profiles (PCC>0.9) (Figure S2B), suggesting that NNML_np_ has overfitted the mixed profiles when there is not a new population in the mixed samples. In contrast, results of PERT (Figure S1B) with the whole genome expression profiles were as accurate as the published results in [1]. Figure S3 shows that expression values of 73.76% of the whole genome were not significantly perturbed (0.8<ρ<1.25), suggesting that PERT was also applicable to circumstances at which heterogeneous samples were physical mixtures of reference populations. We suspect that PERT may have compensated for gene expression changes due to intensity saturation effects or due to RNA-RNA interactions.

Figure S1. PERT recovers composition of known heterogeneous samples. (A) Results of NNML_np_ with the whole genome expression profiles. The percentages were obtained by normalizing the model-predicted proportions for the 4 cell lines to sum to 100% after removing the proportions of the new population. (B) Results of PERT with the whole genome expression profiles.


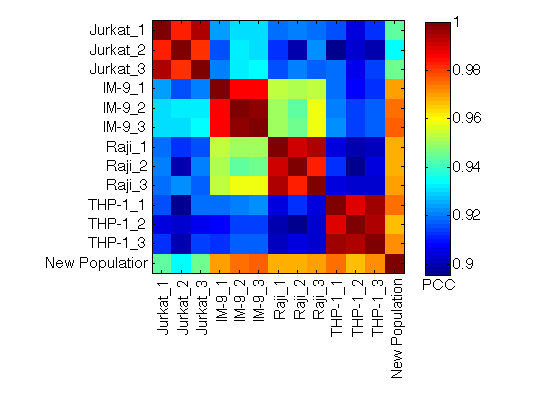


**A**

**B**

Figure S2. NNML_np_ can mistakenly predict the significant presence of a new population of cells. (A) Deconvolved proportions of a new population in mixtures of RNA samples of four cell lines. (B) Pearson correlation coefficients between the model-predicted new population profile and the reference profiles.

Figure S3. Expression of 73.76% of the whole genome was not perturbed when PERT was used to deconvolve mixtures of RNA samples of four cell lines for which there were no differences between the constituent and the reference profiles.

1. **Experimental protocols for collecting in vivo- and culture-derived CFU-M and megakaryocytes**

Day-0 CD34^-^CD45^-^CD61^+^CD41^+^ megakaryocytes and CD34^-^CD33^+^CD13^+^ colony forming unit-monocyte (CFU-M) are sorted from uncultured human umbilical cord blood mono-nucleated cells. Lineage-depleted cells were sorted from the same mono-nucleated cell sample, and cultured for 4 days in a serum-free condition supplemented with SCF, TPO and FLT3LG. Megakaryocytes and CFU-M were sorted from the culture-derived samples using the same markers as for the day-0 samples. The sorting plots are shown in Figure S4.

Figure S4. Sorting plots for CFU-M and megakaryocytes. (A) Sorting plots for day-0 (left) and day-4 (right) CD34^-^CD45^-^CD61^+^CD41^+^ megakaryocytes. (B) Sorting plots for day-0 (left) and day-4 (right) CD34^-^CD33^+^CD13^+^ CFU-M. Red lines: unstained controls. Blue lines: samples.

1. **Efficiency of PERT in capturing cell culture effects in gene expression profiles**

We applied hierarchical clustering to the original and perturbed profiles of uncultured samples (called original and perturbed day-0 profiles, respectively, in the following text) and the original profiles of culture-derived samples (called day-4 profiles in the following text). The day-4 profiles clustered together (Figure S5), suggesting that the culture effect still existed after correction by the perturbation factor. However, the perturbed day-0 profiles clustered closer to the day-4 profiles than to the original day-0 profiles, suggesting that the genome-wide perturbation factor has captured some of the systematic differences between day-0 and day-4 profiles of the same cell type. We suspect that in order to achieve cell type-specific clusters, cell type-specific perturbation factors are required, an approach we did not implement. This model would be similar to an expression deconvolution model in which both the reference populations and their proportions were unknown except that there would be a strong prior to guide the deconvolution. We suspect that such model would require more data to fit.

We attribute the improved performance of PERT to its more appropriate assumptions than the other models for our deconvolving samples. Comparing the day-0 and day-4 profiles (Figure S6A and Figure S6C), expression levels of the highly expressed probes in day-0 samples were more conserved in day-4 samples than the poorly expressed probes. We thus hypothesized that those highly expressed probes in day-0 samples represented phenotype-associated genes whose expression levels should not change even under different developmental conditions. To test the hypothesis, we ranked the probes of day-0 megakaryocytes based on their expression levels and defined the top 1% of the probes as the megakaryocyte phenotype-associated probes (true positives). Using PERT, we obtained probe-specific multiplicative perturbation when compare day-4 megakaryocyte profiles to day-0 megakaryocyte profiles. Given perturbation thresholds between 1 and 65 (i.e., expressed or repressed by 1 – 65 folds in day-4 megakaryocyte comparing to day-0 megakaryocyte), we compared the probes within the individual perturbation threshold and the megakaryocyte phenotype-associated probes. Figure S6B shows that the probes with low perturbation (either repressed or stimulated by <2-fold in day-4 megakaryocyte) were more predictive for the megakaryocyte phenotype-associated probes. The same conclusion was reached for CFU-M (Figure S6D). Superior performance of PERT to NNML and NNML_np_ is likely because PERT has preserved the expression profiles of phenotype-associated probes while taking care of the systematic effects of environmental factors on the whole genome expression.

Figure S5. PERT captures some systematic gene expression differences between day-0 and day-4 samples. Hierarchical clustering of the original and perturbed day-0 profiles and the day-4 profiles. The clusters were calculated from log_2_ gene expression profiles using Pearson’s correlation coefficients as the similarity metric and average-linkage for node summarization.

Figure S6. Expression of highly expressed genes in day-0 megakaryocyte and CFU-M were conserved in megakaryocyte and CFU-M enriched from hematopoietic stem and progenitor cell (HSPC) expansion culture. (A) Gene expression values of day-0 megakaryocytes versus day-4 megakaryocytes derived from human HSPC expansion culture. The data points in red are megakaryocyte phenotype-associated probes defined as the top 1% highly expressed probes. (B) Receiver operating characteristic (ROC) of using probes with perturbation factors less than or equal to a given perturbation level, ranging from 1 to 65, to predict the megakaryocyte phenotype-associated probes. (C) Gene expression values of day-0 CFU-M versus day-4 CFU-M derived from human HSPC expansion culture. The data points in red are CFU-M phenotype-associated probes defined as the top 1% highly expressed probes. (D) ROC of using probes with perturbation factors less than or equal to a given perturbation level ranged between 1 and 65 to predict CFU-M phenotype-associated probes. MEGA: megakaryocyte. CFU-M: colony-forming unit-monocyte.

1. **Reference profiles for decomposing uncultured umbilical cord blood samples**

Gene expression profiles of 21 human umbilical cord blood-derived pure populations (Table S5) from [2] were used as the reference profiles. The 21 pure populations represent 11 blood developmental and functional lineages, namely primitive progenitor cells (PPC), common myeloid progenitors (CMP), megakaryocyte-erythroid progenitors (MEP), megakaryocytes (MEGA), erythrocytes (ERY), granulocyte-monocyte progenitors (GMP), granulocytes (GRAN), monocytes (MONO), basophils (BASO), eosinophils (EOS), and precursor B cells (PREB).

1. **Performance of NNML_np_ and PERT in decomposing mixed samples for which reference populations are incomplete in representing potential constituent populations**

We applied NNML_np_ and PERT to the uncultured lineage-depleted (Lin-) samples and mono-nucleated cell (MNC) samples enriched from human umbilical cord blood data. For NNML_np_, Figure S7 shows that the proportions of new populations were nearly 0% and 100% for the Lin- samples and MNC samples, respectively. The 0% matches our expectation because the major constituents of Lin- samples are in the reference profiles for deconvolution. Also, a new population is unlikely to be present because the mixed samples and the reference samples were both uncultured. However, expression profiles of constituents of MNC samples, such as T cells, B cells, NK cells and dendritic cells, were not in the reference profiles because we did not have gene expression data for those cells enriched from human umbilical cord blood. These results suggested that NNML_np_ was robust for predicting the proportion of a new population in biological samples. For PERT, 83.2% of the whole genome have undergone 0.8- to 1.25-fold perturbation for the MNC samples. The genes with large perturbation (>2-fold) were enriched in immune system process (Table S8), suggesting that the perturbation factor has captured unexplained signals of mixed profiles due to missing reference profiles.

Figure S7. Deconvolved proportions of new populations in uncultured lineage depleted (Lin-) and mono-nucleated cell (MNC) samples enriched from human umbilical cord blood.

1. **Reference profiles for decomposing culture-derived lineage-depleted samples**

Gene expression profiles of 20 reference populations [2], excluding the PPC marked by CD34^+^CD38^-^ antigen expression were used as reference profiles (Table S5). Cell surface marker combination CD34^+^CD38^-^ defines uncultured human blood primitive progenitors; cell colony-forming ability and ability to reconstitute the hematopoietic system after transplantation have been associated with the expression of this cell surface marker combination. However, almost 100% of cells are CD34^+^CD38^-^ on the 12^th^ day of a serum free hematopoietic stem and progenitor cell expansion culture as measured by flow cytometry (data not shown). Furthermore, for culture-derived cells, expression of CD34^+^CD38^-^ does not associate with the colony-forming ability (Figure S8) or the ability to reconstitute the hematopoietic system, as for uncultured cells, after transplantation [3-5]. Thus, the gene expression profiles of CD34^+^CD38^-^ PPCs were excluded from reference profiles for the analysis of culture-derived samples.


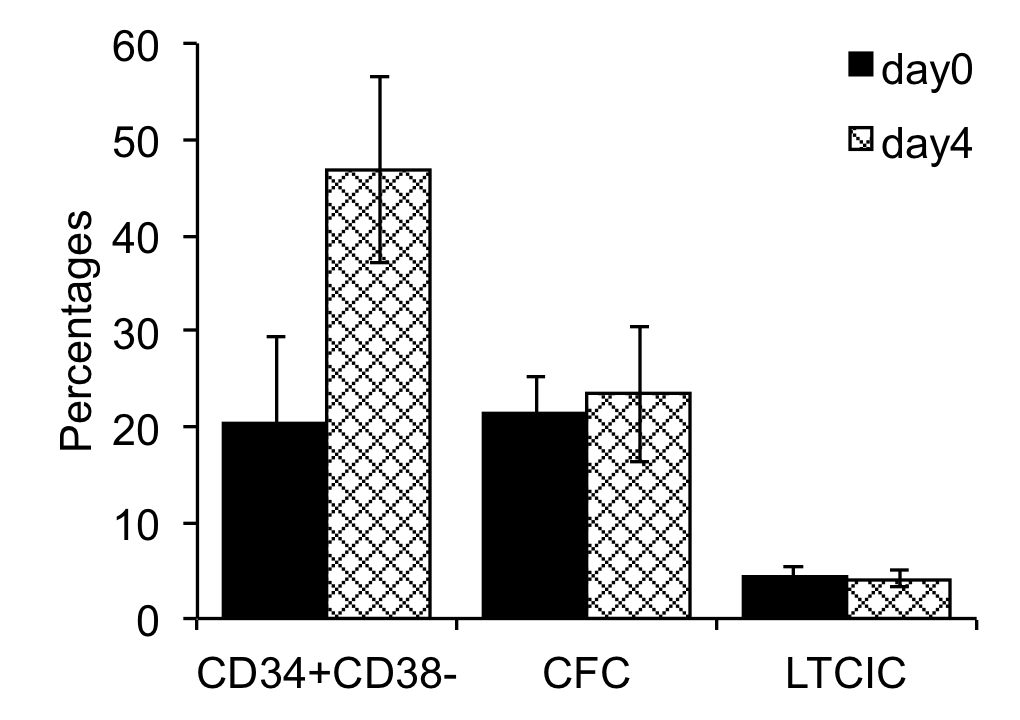


**Figure S8.** Frequencies of CD34^+^CD38^-^ cells, colony forming cells (CFCs) and long-term colony-initiating cells (LTC-IC) within uncultured (day-0) human umbilical cord blood lineage-depleted cells, and in culture-derived (day-4) lineage-depleted cells.

1. **Applicability of NNLS and PERT in decomposing blood cell samples**

In the hematopoietic system, mature cells are generated from hematopoietic stem and progenitor cells through an amplifying differentiation hierarchy. Expression of critical transcription factors or genes, which are largely restricted to specific lineages, increases sequentially from hematopoietic stem cells to definitive lineage progenitors, and to a terminally differentiated population. Identifying a set of transcription factors and genes that distinguishes all the cell types with the system is challenging and is an area under active investigation [2,6]. Application of NNLS is thus inhibited when proportions of individual cell types within the whole system are interested, and deconvolution tools such as PERT which does not require cell type-specific signature transcription factors or genes are desirable.

We compared performance of NNLS and PERT using only the expression of signature genes when the models were used to deconvolve human umbilical cord blood samples (for which compositions were not pre-specified). Signature genes of 3 major hematopoietic lineages – hematopoietic stem and progenitor (HSPC, including primitive progenitors and early erythrocytes), differentiated erythrocytes (ERY) and granulocytes/monocytes (GM, including GMP, GRAN, MONO, EOS and BASO) – were obtained from [2]. Methods for identifying the differentially over-expressed genes are described in the original paper. As NNLS requires an optimal number of signature genes per reference population to be accurate [1], genes of individual lineages were ranked based on Z-scores obtained from [2]; and then condition numbers of matrices composed of expression values of the top N (N = {25, 30, 35…400}) genes of each lineage was computed. The matrix constructed from the top 40 genes of each lineage had the largest condition number and thus was used with NNLS and PERT. Performance of NNLS and PERT were compared. Figure S9 and Table S1 show that performance of PERT was inferior to that of NNLS when cell lineage signature genes were used.

**Figure S9.** Performance of PERT was inferior to NNLS when the models were used with signature genes of blood cell lineages. (A) Flow cytometry measured percentages of GM, ERY and HSPC in uncultured Lin- samples (red) and MNC samples (blue). (B) NNLS deconvolved percentages of GM, ERY and HSPC in uncultured Lin- samples (red) and uncultured MNC samples (blue). (C) PERT deconvolved percentages of GM, ERY and HSPC in uncultured Lin- samples (red) and uncultured MNC samples (blue). (D) Flow cytometry measured percentages of GM, ERY and HSPC in Lin- samples derived from HSPC expansion culture (day-4). (E) NNLS deconvolved percentages of GM, ERY and HSPC in Lin- samples derived from HSPC expansion culture (day-4). (F) PERT deconvolved percentages of GM, ERY and HSPC in Lin- samples derived from HSPC expansion culture (day-4). HSPC: hematopoietic stem and progenitor cells, including primitive progenitors and early erythrocytes. ERY: differentiated erythrocytes lineage. GM: granulocyte/monocyte lineage, including GMP, GRAN, MONO, EOS and BASO. Lin-: lineage-depleted cells. MNC: mono-nucleated cells. The signature genes were obtained from [2].

**Table S1.** R^2^ and averaged absolute differences between model predicted compositions and FACS measured compositions when NNLS and PERT were used with signature genes of 3 blood cell lineages, hematopoietic stem and progenitor cells, erythrocytes, and monocyte/granulocytes obtained from [2].

**References**

1. Abbas AR, Wolslegel K, Seshasayee D, Modrusan Z, Clark HF (2009) Deconvolution of blood microarray data identifies cellular activation patterns in systemic lupus erythematosus. PLoS ONE 4: e6098. doi:10.1371/journal.pone.0006098.

2. Novershtern N, Subramanian A, Lawton LN, Mak RH, Haining WN, et al. (2011) Densely interconnected transcriptional circuits control cell States in human hematopoiesis. Cell 144: 296–309. doi:10.1016/j.cell.2011.01.004.

3. Dorrell C, Gan OI, Pereira DS, Hawley RG, Dick JE (2000) Expansion of human cord blood CD34(+)CD38(-) cells in ex vivo culture during retroviral transduction without a corresponding increase in SCID repopulating cell (SRC) frequency: dissociation of SRC phenotype and function. Blood 95: 102–110.

4. Kusadasi N, Koevoet JL, van Soest PL, Ploemacher RE (2001) Stromal support augments extended long-term ex vivo expansion of hemopoietic progenitor cells. Leukemia 15: 1347–1358.

5. Laer von D, Corovic A, Vogt B, Fehse B, Roscher S, et al. (2000) Loss of CD38 antigen on CD34+CD38+ cells during short-term culture. Leukemia 14: 947–948.

6. Notta F, Doulatov S, Poeppl A, Jurisica I, Dick JE (2010) Isolation of single human hematopoietic stem cells capable of long-term multilineage engraftment.
