# Supplementary material for: PERT: A Method for Expression Deconvolution of Human Blood Samples from Varied Microenvironmental and Developmental Conditions
Source: PLoS Comput Biol. 2012 Dec 20;8(12):e1002838. doi: 10.1371/journal.pcbi.1002838 (PMC3527275; doi:10.1371/journal.pcbi.1002838)
Supplement: Text S2 — Initialization and usage of NNML, NNMLnp and PERT. (DOCX) [file pcbi.1002838.s018.docx]

**Text S2:**

**PERT: a method for expression deconvolution of human blood samples from varied microenvironmental and developmental conditions**

Wenlian Qiao, Gerald Quon, Elizabeth Csaszar, Mei Yu, Quaid Morris, Peter W. Zandstra

1. **Initialization of NNML_np_ parameters**

The hidden variable ***α*** of NNML_np_ was initialized such that each entry of the vector was set to a random number drawn uniformly from [1,2]. The last entry of the vector (corresponding to the unknown cell population) was then further incremented by 5. The parameter *κ* was initialized to the maximum of either 10^4^, or 10/minimum(***β***). The hidden variables ***θ****_d_* were initialized such that 50% of each heterogeneous sample was attributed to the new population, and the remaining fraction of each sample was distributed evenly among the remaining populations. The model parameter ***ω*** was initialized such that all entries of the vector were 1/K. The hidden variable ***γ*** was initialized to the value ***ω***^T^***β***.

1. **Initialization of PERT parameters**

The hidden variable ***α*** of PERT was initialized such that each entry of the vector was set to one more than a random number drawn uniformly from [1,2]. The parameter *κ* was initialized to 1. The hidden variables ***θ****_d_* were initialized such that each component was assigned a random number drawn uniformly from [0,1], then all numbers re-scaled to sum to one. The model parameter ***ρ****_g_* was all initialized to one.

1. **Deconvolution protocol**

The NNML, NNML_np_ and PERT were written in Octave. Two inputs are required for each model: one matrix contains the heterogeneous profiles with genes as rows and samples as columns, and the other matrix is the reference profiles with genes as rows and samples as columns. Elements of the output vector ***θ*** are fractions of mixed profiles attributed to each reference profiles. In order to get the fraction of reference population *i* that has N*_i_* replicates of gene expression profiles, deconvolved *θ*’s for the N*_i_* replicates are summed.

NNML

>> load(‘gene_expression_data.mat’)

>> addpath(genpath(‘directory_of_NNML’))

>> [nnml, loglikelihood] = directoptlearn(MixedProfiles, transpose(ReferenceProfiles));

NNML_np_

>> load(‘gene_expression_data.mat’)

>> addpath(genpath(‘directory_of_NNML_np_’))

>> [nnml_np, loglikelihood] = directoptlearn(MixedProfiles, transpose(ReferenceProfiles), transpose(ReferenceProfiles));

For a data set consisted of X reference profiles, the output ***θ*** vector has X+1 elements. The last element is the deconvolved proportion of a new population. In order to get the actual fractions of the X reference profiles to the heterogeneous sample, θ values for the X reference profiles are renormalized to sum up to 1.

PERT

>> load(‘gene_expression_data.mat’)

>> addpath(genpath(‘directory_of_PERT_model’))

>> [pert, loglikelihood] = directoptlearn(MixedProfiles, transpose(ReferenceProfiles));
